# Temporal disorientation during war: associations with work-family conflict, emotional distress, and burnout

**DOI:** 10.3389/fpsyg.2025.1565639

**Published:** 2025-05-20

**Authors:** Irene Diamant, Maor Kalfon Hakhmigari

**Affiliations:** Academic College Tel Aviv-Yaffo, Yaffo, Israel

**Keywords:** temporal disorientation, emotional distress, burnout, work-family conflict, war

## Abstract

**Introduction:**

Disruptions in the temporal experience—such as subjective sense of time being long, short or distant compared to “objective” time—have been found in the context of extreme emotional events. This research, conducted a month after the start of the war in Israel, aimed to identify characteristics of temporal experience among a population facing ongoing crisis conditions, and to examine their relationship to emotional distress and burnout. Based on Hobfoll's Conservation of Resources Theory it was hypothesized that temporal disorientation is essentially the loss of a vital psychological-functional resource, and its intensity would be related to emotional distress and burnout during the war period.

**Methods:**

The study involved 374 participants, recruited using a snowball sampling technique. They completed an online survey aimed at quantifying Temporal Disorientation, Burnout, Psychological Distress and Work-Family Conflict. Principal Component Analysis (PCA) was used to validate the factor structure of the Hebrew version of Temporal Disorientation questionnaire, Pearson correlation coefficient was used to evaluated correlations among the study variables; Hierarchical linear regression was used to model the outcome variables, emotional distress and burnout.

**Results:**

Temporal confusion and difficulties in future orientation during wartime were significantly associated with emotional distress. The findings also indicate that temporal confusion and difficulties in future orientation were related to a loss of balance in the boundaries between major life roles, work-family conflict, and the development of occupational burnout.

**Discussion:**

This study contributes an occupational angle to the existing literature on psychological reactions to prolonged crises. Understanding the role of temporal experience during a prolonged crisis can significantly contribute to intervention and prevention measures in the context of the development of mental pathology. Practical implications and study limitations are discussed.

## 1 Introduction

Continuity and structure in the experience of time are essential for mental health, identity cohesion, and stable functioning. A sense of time as continuous and systematic—from past to future—enables interpretation, prediction, planning, and control. “Objective” time, shared and precise, follows a steady, linear progression. In contrast, “psychological” time, the subjective internal experience, can deviate from this regular flow.

Disruptions in the temporal experience—such as a subjective sense of time being long, short, fragmented, or distant compared to “objective” time—have been found in the context of experiencing extreme emotional events, boredom, waiting, fear, pain, and threat (Droit-Volet et al., 2020; Gil and Droit-Volet, 2012; Grommet et al., 2011; Grondin et al., 2020; Ogden et al., 2024; Zakay, 2014). Such disruptions have also been documented in relation to various psychopathological disorders, especially depression, anxiety, and post-trauma (Holman and Grisham, 2020; Kosak et al., 2022; Maiese, 2018; Thönes and Oberfeld, 2015; Treitel and Levy-Gigi, 2023). Specifically, research has shown that ongoing crisis, like the COVID-19 pandemic, was linked to temporal disorientation: confusion in time, a sense of prolonged and slow time, and difficulty in relating to the future (Ogden, 2021; Pawlak and Sahraie, 2023; Taub et al., 2022; Fernandez Velasco et al., 2022). Studies also indicated a correlation between temporal disorientation and emotional distress during and after the crisis (Droit-Volet et al., 2023). The current research was conducted about a month into the war in Israel, which began on October 7, 2023, severely disrupting routine life and existential security. The study aimed to identify characteristics of temporal experience during this crisis and examine their relationship to emotional distress and burnout.

Periods of crisis, especially wartime, are marked by heightened emotional distress and mental health decline. A meta-analysis found significantly higher rates of depression and anxiety in conflict zones compared to post-war periods (Lim et al., 2022). This pattern has also been observed in the context of the current war in Israel, with recent studies documenting elevated levels of post-traumatic stress, anxiety, and depression among various segments of the population (Bergman et al., 2025; Levi-Belz et al., 2024; Saar-Ashkenazy et al., 2024). While psychological distress during war is well-documented, workplace wellbeing, particularly burnout, is often overlooked. The WHO defines burnout as a syndrome from “chronic workplace stress that has not been successfully managed,” characterized by exhaustion, mental detachment, and reduced efficacy (World Health Organization, 2019; Maslach et al., 2001). Few studies explore burnout during wartime, but examples include 67% of Libyan healthcare workers reporting high burnout, particularly emotional exhaustion, and 61% of female and 48% of male Ukrainian academic staff experiencing severe emotional fatigue and impaired academic functioning (Tsybuliak et al., 2023).

### 1.1 The temporal experience as a resource: a conservation of resources theory perspective

Understanding mental health during crises can be framed through the lens of the Conservation of Resources (COR) theory. The theory suggests that individuals are driven by the need to acquire, build, develop, and protect resources that they value (Hobfoll, 1989). Such resources can include a sense of security, stable livelihood, and a sense of self-efficacy. According to this approach, emotional distress, including burnout, develops as a result of a prolonged process of resources loss.

COR theory provides a foundation for understanding emotional responses to resource loss in large-scale crises like pandemics, wars, disasters, and terrorism (Hobfoll, 2001; Hobfoll et al., 2006, 2012; Ironson et al., 1997; Smith and Freedy, 2000). Studies indicated a relationship between resources loss and emotional distress following traumatic events like hurricanes (Sattler et al., 2002), 9/11 terrorist attack in New York (Galea et al., 2002), and COVID-19 (Yu et al., 2023). Research also highlights that wartime resources loss—such as personal security, stability, economic, and social supports, trust and hope —significantly increases the risk of post-traumatic symptoms (Dekel and Hobfoll, 2007; Hobfoll et al., 2006, 2011).

Despite extensive research demonstrating the relevance of the COR theory to explaining various aspects of emotional distress during crises, the mechanisms of resources loss remain underexplored (Toker et al., 2015). The current study investigates whether temporal disorientation, as a significant loss of resources, is associated with emotional distress and burnout.

### 1.2 Disruptions in the temporal experience

Psychological time refers to how we perceive, experience, or evaluate time, contrasting with “objective” or “clock” time (Block and Zakay, 2000; Fraser, 1966; James, 1890). This subjective experience shapes daily life, aiding adaptation, continuity, and identity (McAdams, 2001; Prebble et al., 2013). Bergson (1946) emphasized subjective time as central to self-consciousness, describing it as “real” time—a memory-based continuity where the past persists in the present.

Disruptions occur when subjective time diverges from objective time, manifesting as altered perceptions of time passing (faster or slower) or shifts in temporal distances (e.g., feeling much or little time has passed since an event) (Fernandez Velasco et al., 2022). These disruptions reflect the interplay between environmental factors and mental processes and are linked to depression, anxiety, and trauma (Holman and Grisham, 2020; Thönes and Oberfeld, 2015). Negative emotions, in particular, are associated with a sense of time slowing down (Gil and Droit-Volet, 2012; Ishikawa and Okubo, 2016; Ogden, 2021).

During the COVID-19 pandemic, many reported a sense that time was passing either more slowly or quickly than usual (Cellini et al., 2020; Droit-Volet et al., 2023), with higher levels of depression linked to the perception that more time had passed since the pandemic began (Ogden and Piovesan, 2022). Another aspect of temporal disruption is future orientation, which is crucial for focusing on goals, fulfilling aspirations, and making plans (Baumeister et al., 2016; Zimbardo and Boyd, 1999). In times of crisis, such as war, difficulties in predicting or imagining the future, or a lack of control over it, have been shown to predict emotional distress, as well as motivational and behavioral challenges (Andre et al., 2018; Gamble et al., 2019).

Fernandez Velasco et al. (2022) developed a tool for assessing aspects of temporal disruptions and disorientation during the COVID-19 pandemic and identified six measures: *disruptions in passage of time (PT)—*a sense that time is passing more slowly or quickly than usual, *disruptions in temporal order of events (TOE)—*memory for the correct order in which events occurred, *disruptions in temporal distance (TD)*—an intuitive sense of closeness or distance in time from a certain point in time), *disruptions in future orientation (FO)*—mental reference to the future, *disruptions in temporal self-location (TSL)—*knowledge of the current hour, day, month, and *disruption in temporal rupture (RT)—*a sense of temporal continuity being interrupted by a sudden severe event.

Fernandez Velasco et al. (2023) found that the COVID-19 pandemic was characterized by temporal disorientation, with people experiencing anomalies in time perception, temporal distance, sequence, orientation, and future reference. They also discovered correlations between these dimensions: individuals who felt time was slower also experienced confusion about event order and greater temporal distance. Slower time perception was linked to social disorientation and increased trauma responses. The authors suggested their tool could be useful for studying time perception during crises that disrupt normal temporal markers, highlighting the importance of exploring its connections to other psychological phenomena.

### 1.3 Work-family conflict

Work and family, as two major roles in an individual's life, form the foundation of their daily routine, shaping the rhythm of the day and their experience of time. Work-family conflict is typically defined as a form of inter-role conflict where participation in one role (work or family) is hindered by involvement in the other (Greenhaus and Beutell, 1985). This conflict can occur in both directions: when work interferes with family life (work-to-family conflict) or when family responsibilities disrupt work (family-to-work conflict) (Frone, 2003). Higher levels of work-family conflict were associated with greater psychological and physical health problems (e.g., Allen et al., 2000). Recent research emphasizes the dynamic relationship between work and family roles across temporal changes, through both life cycle stages, everyday events, and major events (Allen et al., 2023; Piszczek and Yestrepsky, 2024). Boundary theory helps to explain how individuals establish and manage boundaries between work and family roles to effectively organize their time and resources. As the dynamic relationship between these roles evolves, these natural boundaries have increasingly blurred (Ashforth et al., 2000). Despite this, there is still limited knowledge about how this conflict develops across different temporal changes (Allen et al., 2023). The blurring of boundaries may be further exacerbated by acute life events. For instance, during the COVID-19 pandemic, higher levels of work-family conflict were observed (Barriga Medina et al., 2021).

### 1.4 The current study

The primary aim of the current study was to examine temporal disorientation during wartime. It was hypothesized, similar to Fernandez Velasco et al. (2023), that disorientation would manifest in various dimensions, and correlations would be found between these dimensions and the level of emotional distress and burnout during the crisis. Additionally, it was hypothesized that disruptions in time would have significant correlation with a psychological phenomenon sensitive to temporal structure and order: work-family conflict. Finally, the study examined how components of temporal disorientation and work-family conflict together relate to burnout and emotional distress.

## 2 Materials and methods

### 2.1 Participants

The study included 374 participants (261 women, 112 men, 1 unreported gender), aged 28 to 60. Of these, 271 (72.5%) were married or cohabiting, and 314 (84%) had higher education. All were employed, and none were in military service or evacuated due to the war. Conducted 1 month after the war began, participants were asked if they or their close ones had been physically injured by terror or war. Four participants (1.1%) reported personal injuries, while 37 (17.1%) reported injuries to friends or relatives.

### 2.2 Recruitment and procedure

The study was conducted from November 7 to November 19, 2023, 1 month after the war began. Ethical approval was obtained from the Institutional Review Board (IRB) of The Academic College of Tel-Aviv Yaffo. Participants were recruited via snowball sampling on social media and completed the survey on Qualtrics (http://www.qualtrics.com). They were invited to participate in a survey on coping with the war in Israel, with assurances of anonymity and voluntary participation. Informed consent was obtained, and participants were free to withdraw at any time. Researchers' contact information was provided.

### 2.3 Measures

#### 2.3.1 Sociodemographic questionnaires

Sociodemographic questionnaires included gender, age, family status, and education level. Additionally, participants were asked about physical injuries due to war.

#### 2.3.2 Temporal disorientation

Temporal disorientation was assessed using the Instrument for Measuring Temporal and Social Disorientation (ITSD) developed by Fernandez Velasco et al. (2022). The instrument consists of 18 items related to six components of temporal disorientation: Temporal Order of Events (TOE), Temporal Self Location (TSL), Passage of Time (PT), Future Orientation (FO), Temporal Distance (TD), and Temporal Rupture (TR). We translated the questionnaire to Hebrew, using forward-backward translation by the researchers (Brislin, 1970), and principal component analysis (PCA) was used to validate the factor structure of the Hebrew version. Parallel analysis was based on the correlation matrix with Promax oblique rotation to account for inter-component non-orthogonal relationships. The Kaiser-Meyer-Olkin (KMO = 0.70) and Bartlett's test [χ^2^(153) = 1,576.93, *p* < 0.001] confirmed the suitability of the data for analysis.

The results revealed a factor structure that was similar yet somewhat different from the original. Fifteen items loaded onto four factors. Items 3 and 4 of the original TSL factor, along with items 5, 6, and 7 from the original TOE factor, loaded onto a new factor, which we named the Temporal Confusion (TC) factor (13.7% variance). An example item: “I get confused more/less often about which day of the week it is.” The three items from the original PT factor remained unchanged (12.8% variance). An example item: “At times, since the war began, time has been passing noticeably slowly.” The three items from the original FO factor with the addition of item 17 (originally belonged to the TR factor, formed the third factor (FO+TR17; 11.7% variance). An example item: “I feel it is easier/harder for me to imagine the future.” Finally, the original TD factor retained its factor structure perfectly as the fourth factor (9.9% variance). An example item: “At times, the beginning of the war feels noticeably far away.” Together, these four factors accounted for 48.1% of the total variance. Component loadings are provided in Table 1.

**Table 1:** The instrument for measuring temporal disorientation's component loadings (Hebrew version).

**Item**	**1**	**2**	**3**	**4**	**Uniqueness**
	**TC**	**PT**	**FO**	**TD**	
(5) At times, I feel confused about the order of events that occurred since the war began (TOE)	0.79				0.37
(4) I get confused more/less often about which month of the year it is (TSL)	0.75				0.50
(7) I feel it is easier/harder to imagine for me to recall events that have taken place since the war began (TOE)	0.64				0.60
(6) At times, I feel confused about the order of events that occurred before the war began (TOE)	0.62				0.52
(3) At times, I feel confused about the order of events that occurred before the war began (TSL)	0.49				0.61
(13) Overall, since the war began, time has been passing slowly/quickly (PT)		0.90			0.18
(12) At times, since the war began, time has been passing noticeably quickly (PT)		0.89			0.24
(11) At times, since the war began, time has been passing noticeably slowly (PT)		0.73			0.30
(14) I feel it is easier/harder for me to imagine the future (FO)			0.82		0.38
(15) I feel I'm more/less anxious about my future (FO)			0.78		0.42
(16) I feel I'm more/less in control of my future (FO)			0.57		0.63
(17) At times, the period since the war began felt unreal to me (TR)			0.45		0.66
(8) At times, the beginning of the war feels noticeably far away (TD)				0.83	0.26
(10) Overall, times before the war feel as if they are further away/closer to me than they really are (TD)				0.75	0.38
(9) At times, the beginning of the war feels noticeably close (TD)				0.43	0.61
(1) I feel I'm more/less reliant on calendars or to-do lists to keep track of what I do (ATO)					0.96
(2) I care more/less about following a routine (daily, or weekly) (ATO)					0.82
(18) The period since the pandemic began feel connected/disconnected from the months and years prior (TR)					0.88

Applied rotation method is Promax. Only loadings above 0.4 are displayed. In parenthesis next to item number is the original factor as reported by Fernandez Velasco et al. (2022). TOE, Temporal Order of Events; TSL, Temporal Self Location; PT, Passage of Time; FO, Future Orientation; TD, Temporal Distance; TR, Temporal Rupture; ATO, Assistance in Temporal Orientation.

The factors were transformed into new study variables by averaging their five-point Likert scores. The Cronbach's alpha reliabilities were satisfactory to good as follows: TC = 0.70, PT = 0.83, FO = 0.61, and TD = 0.66.

#### 2.3.3 Occupational burnout

Occupational burnout was assessed using the Hebrew version of the Shirom-Melamed Burnout Measure (SMBM), a 14-item scale evaluating job burnout. Participants reported how often they experienced physical fatigue, cognitive weariness, and emotional exhaustion at work over the past month, with responses ranging from 1 (almost never) to 7 (almost always). The mean score across all items was calculated (Shirom and Melamed, 2006). The SMBM showed strong internal consistency with a Cronbach's alpha of 0.94.

#### 2.3.4 Emotional distress

Emotional distress was assessed using the Hebrew version of the Depression, Anxiety, Stress Scales (DASS-21), a 21-item self-report questionnaire evaluating symptoms of depression, anxiety, and stress over the past week. Each subscale includes seven items, with responses rated on a 4-point Likert scale from 1 (did not apply to me at all) to 4 (applied to me very much) (Lovibond and Lovibond, 1995; Lurie-Beck et al., 2008). The DASS demonstrated strong internal consistency with a Cronbach's alpha of 0.95.

#### 2.3.5 Work-family conflict

Work-family conflict was assessed using an eight-item measure developed for the MIDUS study by Allen et al. (2023). Four items measured work-to-family conflict and four measured family-to-work conflict. Responses were rated on a 5-point Likert scale from 1 (Never) to 5 (All of the time). The questionnaire was translated into Hebrew using forward-backward translation (Brislin, 1970). Both work-to-family and family-to-work conflict demonstrated strong internal consistency, with Cronbach's alphas of 0.82 and 0.76, respectively.

### 2.4 Statistical analysis

Statistical analysis included Pearson correlations among study variables and Principal Component Analysis to examine the factor structure of temporal disorientation. Hierarchical linear regression modeled emotional distress and occupational burnout. In Step 1, gender and temporal disorientation components were included; in Step 2, work-to-family and family-to-work conflicts were added. Results are presented with standardized (β) and unstandardized (B) coefficients, standard errors (SE), *p*-values, and adjusted explained variance (R^2^). A significance level of *p* < 0.05 was used. Data analysis was performed using SPSS version 28.0.

## 3 Results

Table 2 shows Pearson correlations among the research variables, identifying several significant relationships. Greater temporal confusion (TO) was related to weaker future orientation (FO) and increased work-to-family and family to work conflict. Perceiving time as faster (PT) makes events feel closer (TD). Higher emotional distress was associated with increased burnout and both sides of work-family conflict. Additionally, slight gender differences emerged, with women reporting higher levels of temporal confusion and burnout.

**Table 2:** Pearson correlations between the research variables.

**Variable**	***M***	***SD***	**TC**	**PT**	**FO**	**TD**	**WFC**	**FWC**	**DASS**	**BO**
Temporal confusion	2.99	0.69								
Passage of time	2.91	0.96	0.05							
Future orientation	3.70	0.60	−0.26^**^	−0.08						
Temporal distance	2.46	0.92	0.30^**^	0.22^**^	−0.13^**^					
Work–to–family conflict	2.87	0.83	−0.26^**^	−0.02	0.27^**^	−0.12^*^				
Family–to–work conflict	2.60	0.77	−0.33^**^	−0.07	0.34^**^	−0.09	0.55^**^			
Emotional distress (DASS)	0.92	0.59	−0.35^**^	−0.14^**^	0.39^**^	−0.16^**^	0.45^**^	0.42^**^		
Burnout	3.95	1.15	−0.33^**^	−0.10	0.33^**^	−0.18^**^	0.47^**^	0.42^**^	0.62^**^	
Gender			−0.12^*^	−0.12^*^	0.05	−0.10	0.10	0.02	0.11^*^	0.19^**^

* p < 0.05,

** p < 0.01.

Male = 0, female = 1. TC, Higher scores indicate lower levels of temporal confusion.

Regression analysis (Table 3) examined how temporal disorientation and work-family conflict predict emotional distress. Gender and temporal disorientation (TC, PT, FO, TD) explained 23% of emotional distress variance (*R*^2^ = 0.23, *p* < 0.001), with TC negatively and FO positively predicting emotional distress (*B* = −0.24, *p* < 0.01; *B* = 0.31, *p* < 0.001). PT and TD were not significant. Adding both sides of work-family conflict increased explained variance to 34% (*R*^2^ = 0.34, *p* < 0.001), with TC, FO, work-to-family and family-to-work conflict, all being significant predictors.

**Table 3:** Regression model of disorientation components and work and family conflicts on emotional distress.

**Variable**	**Step 1**			**Step 2**		
	***B***	***SE***	β	***B***	***SE***	β
(Constant)	0.64^*^	0.27		−0.09	0.27	
Gender	0.06	0.06	0.05	0.04	0.06	0.04
TC	−0.24^***^	0.05	−0.24^***^	−0.15^**^	0.05	−0.16^**^
PT	−0.05	0.03	−0.09	−0.05	0.03	−0.09
FO	0.31^***^	0.05	0.32^***^	0.22^**^	0.05	0.22^***^
TD	−0.01	0.03	−0.02	−0.01	0.03	−0.02
Family–to–work conflict				0.10	0.04	0.14^*^
Work–to–family conflict				0.19	0.04	0.27^***^
R^2^	0.23^***^			0.34^***^		
R^2^ change				0.11^**^		

* p < 0.05,

** p < 0.01,

*** p < 0.001.

Regression analysis (Table 4) examined how temporal disorientation and both sides of work-family conflict predict burnout. Gender and temporal disorientation (TC, PT, FO, TD) explained 20% of the variance in burnout (*R*^2^ = 0.20, *p* < 0.001), with TC negatively and FO positively predicting burnout (*B* = −0.44, *p* < 0.001; *B* = 0.50, *p* < 0.001). PT and TD were not significant. Adding work-family conflict increased the variance explained to 33% (*R*^2^ = 0.33, *p* < 0.001), with TC, FO, work-to-family and family-to-work conflict all being significant predictors.

**Table 4:** Regression model of disorientation components and work and family conflicts on burnout.

**Variable**	**Step 1**			**Step 2**		
	***B***	***SE***	β	***B***	***SE***	β
(Constant)	3.50	0.54		1.93	0.53	
Gender	0.35^**^	0.12	0.14^**^	0.31^**^	0.11	0.13^**^
TC	−0.44^***^	0.10	−0.23^***^	−0.19^*^	0.08	−0.12^*^
PT	−0.05	0.06	−0.04	−0.05	0.05	−0.04
FO	0.50^***^	0.09	0.26^***^	0.30^**^	0.09	0.16^**^
TD	−0.08	0.06	−0.06	−0.08	0.06	−0.06
Family-to-work conflict				0.25^**^	0.08	0.17^**^
Work-to-family conflict				0.40^***^	0.07	0.29^***^
R^2^	0.20^***^			0.34^***^		
R^2^ change				0.13^**^		

* p < 0.05,

** p < 0.01,

*** p < 0.001.

## 4 Discussion

The current study examined temporal disorientation as a mental-functional mechanism related to work-family conflict, burnout, and emotional distress during an ongoing crisis. The research was conducted at an early stage of a war, characterized by widespread direct effects on the general population in Israel. For the purpose of measuring temporal disorientation, we used a tool developed by Fernandez Velasco et al. (2022), applied for the first time in Hebrew and tested in the context of war. We identified four interrelated components of temporal disorientation (similar yet slightly different from the original reported structure), with *temporal confusion* being central (e.g., confusion about the day, month, or event sequence from the start of the war or before it).

*Temporal confusion* reflects an unraveling of a homogeneous, continuous, stable, and chronological continuity. Participants who experienced higher levels of *temporal confusion* also experienced higher levels of *temporal distance* disorientation (feeling that the start of the war is further away in time than it actually is) and difficulties with *future orientation* (difficulty imagining or controlling the future). In other words, individuals who are “lost in time,” struggling to accurately place the “here and now,” also lose a precise connection to past events (which seem distant) and to the future (which becomes inaccessible, hard to predict, and uncontrollable). These results align with Fernandez Velasco et al. (2023), who also found similar relationships during the COVID-19 pandemic. Similar to the social isolation and dramatic restrictions that existed during the pandemic, war situations create a severe disruption of routine, habits, and stability, contributing to the unraveling of a stable temporal fabric and temporal orientation.

### 4.1 Temporal disorientation, burnout, and emotional distress during war

As expected, the current study found emotional distress a month into the war. However, a key contribution of this study is the identification of emotional distress in the occupational context, including burnout signs such as exhaustion and lack of motivation related to job performance. The connection between emotional distress and burnout was strong (*r* = 0.62, *p* < 0.01). Based on Hobfoll's (1989) Conservation of Resources Theory, we hypothesized that temporal disorientation, as a loss of mental-functional resources, would relate to emotional distress and burnout. In the current study, temporal confusion, and future orientation difficulties were found to predict both burnout and emotional distress during the war.

Our findings on temporal confusion align with literature linking disturbances in temporal experience to depression, anxiety, and trauma during prolonged crises (e.g., COVID-19) (Droit-Volet et al., 2020; Holman and Grisham, 2020; Grondin et al., 2020). The loss of predictability and control, along with the disruption of daily habits, significantly predicts maladaptive psychological reaction to crisis. Struggles to maintain routines during prolonged crises are linked to mental health decline, including depression, anxiety, and poor adaptation (Clay and Greer, 2019; Maugeri et al., 2020; Miller and Rasmussen, 2010; Sherman et al., 2020). Temporal disorientation may arise from these routine disruptions.

Participants with greater difficulty in future orientation (struggling to imagine or feel in control of the future) reported higher levels of burnout and emotional distress. Verfaellie et al. (2023) found similar difficulties among war victims with PTSD. Nowack (2023) suggested that in an unstable risk environment with limited accessible resources, there is more present-focused orientation, less future orientation, and a slower experience of time. He found a significant correlation between the experience of slow time and a lack of future orientation during the COVID-19 pandemic. Baumeister et al. (2016) defined pragmatic prospection as future-oriented thinking that supports practical efficiency, action orientation, promotes goals, and enables desirable outcomes in the future. Their theory of Pragmatic Prospection suggested that a basic premise that future thinking, as a basic mechanism of human existence, is based on the assumption of the ability to act on it. Another premise of the theory is that the future influences present experience by creating meaning. Based on these premises, it can be assumed that a prolonged war crisis, fundamentally characterized by a lack of control, unpredictability, and intense existential anxiety in the affected population, leads to an aversive focus on the present, which in itself loses structure and order, making it difficult to focus on the future. The absence of future orientation, in itself, makes it difficult to find meaning in the present (the present is not serving goals or desirable outcomes), a phenomenon directly related to emotional distress and the development of mental pathology.

### 4.2 Temporal disorientation, work-family conflict, emotional distress, and burnout

The current study highlights a significant association between work-family conflict, burnout, and emotional distress, with a new insight into this relationship during wartime. Furthermore, it identifies a connection between both sides of work-family conflict and temporal disorientation: individuals experiencing temporal confusion and difficulty in future orientation report stronger conflict. No such relationship was observed with the perceived pace of time or temporal distance.

Boundary Theory (Ashforth et al., 2000) suggests that boundary management represents a process in which individuals create, maintain, or modify boundaries in the world they operate in, including regarding their family and work roles. Time and place serve as markers that delineate different roles. For example, the family role is usually associated with home, weekends, and evenings, while the work role is typically associated with weekdays and the workplace. Extreme prolonged crises, such as a pandemic or war, blur such markers, contributing to temporal disorientation. While slow time experience and temporal distance are significantly related in the literature to a negative effective state (depression), temporal confusion, and loss of future orientation may represent a deeper unraveling of time's continuity and coherence. This disintegration is critically related to impairment in functioning and motivation, and in our study, to work-family conflict.

### 4.3 Gender differences

The gender differences observed suggests that women report slightly higher temporal confusion, slower time perception, and greater burnout. These findings could reflect gender-specific challenges in balancing work and family roles, potentially leading to more strain, and burnout among women.

### 4.4 Limitations

This study has some limitations. First, it was conducted at a single time point, therefore limiting the ability to draw causal conclusions. A longitudinal approach could provide a deeper understanding of temporal disorientation's relationship with emotional distress and burnout. In addition, the use of snowball sampling and the focus on a population indirectly affected by the war suggest that broadening the study's scope would be helpful. The sample was also relatively homogeneous, with a high proportion of women and a generally high level of education, which may limit the generalizability of the findings. Future studies should aim to include more diverse populations in terms of gender, educational background, and other demographic factors. Regarding gender differences, is also possible that, in the context of the current war, some women experienced increased responsibilities at home due to their partners' reserve duty. Although only one participant in our sample reported such a situation, future studies should further explore how gendered wartime roles may shape the experience of temporal disorientation and distress.

### 4.5 Implications and further research

The present study underscores the need for advancing theoretical and empirical understanding of the ways in which temporal disorientation relates to psychological functioning and motivation during periods of war, with particular emphasis on occupational contexts and the management of life roles. Such conceptual development holds promise not only for enriching theoretical discourse but also for informing intervention and treatment strategies aimed at sustaining daily routines and enhancing psychological resilience during extended states of crisis.

More broadly, the observed associations between temporal disorientation, emotional distress, and burnout are consistent with a growing body of literature linking disruptions in temporal experience to various forms of psychopathology, particularly depression, schizophrenia, and post-traumatic stress disorder (PTSD). Stanghellini et al. (2017), for instance, conceptualize depression as a disorder of lived time, identifying a deceleration in the subjective experience of time as a core diagnostic feature. Similarly, Blom et al. (2021) have called for further empirical work on the classification of temporal experience anomalies, proposing that temporal phenomenology may serve as a valuable diagnostic marker for specific psychopathological conditions. Future research in this domain may yield significant contributions to diagnostic frameworks.

Furthermore, the finding that diminished future orientation is associated with elevated psychological distress invites both theoretical refinement and practical application within the domain of Time Perspective research. Zimbardo and Boyd (1999) proposed a typology of individual differences in temporal orientation—toward the past, present, or future—demonstrating that these orientations are significantly associated with psychological wellbeing, adaptive functioning, and behavioral regulation. Although initially conceptualized as relatively stable dispositional traits, temporal orientations have also been shown to be modifiable through targeted interventions (Hall and Fong, 2003; Hershfield et al., 2011). Zimbardo and Boyd (2008), for example, suggest that individuals can acquire strategies to overcome maladaptive temporal biases. One intervention rooted in this framework, Time Perspective Therapy, has demonstrated efficacy in the treatment of PTSD (Sword et al., 2014). Continued empirical inquiry in this area may foster the development of additional therapeutic modalities grounded in subjective temporal experience and tailored to individuals facing psychological distress in the context of temporal disorientation.

### 4.6 Conclusions

The current study highlights the importance of the temporal experiential component in the development of emotional distress and burnout during prolonged crises. Szpunar and Liu (2023) discuss the connection between national temporal cognition and individual temporal cognition: the individual temporal experience of past-present-future is intertwined with collective national temporal experience. In this context, crisis events or gross disruptions of life scripts, like in war-time, may amplify such interaction when collective temporal disruption becomes significantly synchronized with individual disruption.

The findings of the current study indicate that temporal disorientation in the individual experience during wartime is significantly associated with emotional distress. The findings also indicate that temporal disruption is related to a loss of balance in the boundaries between major life roles and the development of occupational burnout; thus, this study contributes an occupational angle to the existing literature on psychological reactions to prolonged crises.

The practical implications of these findings point to the importance of maintaining, restoring, or rebuilding a “normal” temporal experience: here and now, time orientation, a continuous sense of time, and connection to the future. Preventing or rehabilitating temporal disorientation during prolonged crises could significantly contribute to the prevention of mental pathology, largely through a sense of predictability, control, influence, and competence.

## Data Availability

The raw data supporting the conclusions of this article will be made available by the authors, without undue reservation.
